# Proanthocyanidins Activate Nrf2/ARE Signaling Pathway in Intestinal Epithelial Cells by Inhibiting the Ubiquitinated Degradation of Nrf2

**DOI:** 10.1155/2022/8562795

**Published:** 2022-08-18

**Authors:** Yang Shuhua, Meng Lingqi, Dang Yunlong, Tang He, Shi Yao, Li Peng

**Affiliations:** College of Animal Science & Veterinary Medicine, Shenyang Agricultural University, Shenyang 110866, China

## Abstract

Nrf2 plays a key role in the antioxidant system, and many antioxidants can activate the Nrf2/ARE signaling pathway and alleviate oxidative stress. However, the underlying mechanisms of antioxidants, such as proanthocyanidin- (PC-) induced Nrf2 activation, remain poorly understood. In this study, PC was used on MODE-K cells at different concentrations (0, 1, 2.5, and 5 *μ*g/mL) and different times (0, 3, 6, 12, and 24 h); then, immunoprecipitation, immunofluorescence, and Western blotting were performed to test Nrf2, Bach1, Keap1, HO-1, and NQO1 protein expressions in MODE-K cells. Results showed that PC increased Nrf2, HO-1, and NQO1 protein expressions, decreased Keap1 and Bach1 protein expressions, and enhanced ARE gene activity. PC also decreased the ubiquitinated degradation of the Nrf2 protein, increased Nrf2 protein stability, and increased Nrf2 protein expression by inhibiting Keap1-dependent Nrf2 protein degradation, promoted Nrf2 entry into the nucleus, competed with Bach1, and activated ARE elements, which in turn initiated the Nrf2/ARE signaling pathway. Thus, we conclude that PC activates the Nrf2/ARE signaling pathway in intestinal epithelial cells by inhibiting the ubiquitinated degradation of Nrf2, increasing Nrf2 protein stability and expression, and then regulating key antioxidant enzymes such as HO-1 and NQO1 to initiate cytoprotective effects.

## 1. Introduction

Animals always experience a variety of exogenous and endogenous harmful substances, such as mycotoxin, heavy metals, and free radicals, which lead to oxidative stress and affect the normal physiological function. Oxidative stress is known to be closely associated with decreased disease resistance and reduced production performance of livestock and poultry. Nuclear factor erythroid-2-related factor 2 (Nrf2)/Kelch-like ECH-associated protein 1- (Keap1-) antioxidant response element (ARE) signaling pathway is one of the most important defense mechanisms against oxidative stress [1]. Under normal physiological conditions, Nrf2 binds to its negative regulatory protein Keap1 and is ubiquitinated and degraded by the ubiquitin-proteasome system [2, 3]. Oxidative stress results in dissociation of Nrf2 from Keap1, and Nrf2 is translocated into the nucleus and recognizes ARE sequence to initiate transcription of downstream target genes, such as SOD (superoxide dismutase) and HO-1 (enzyme heme oxygenase 1) [4–6].

As we known, PC is a natural antioxidant which is mainly rich in tea, blueberry, apples, pears, and grapes [7–9] and has numerous biological activities [8, 10–12]. The gastrointestinal (GI) tract is a complex system that fulfills essential functions in the animal or human body. Many researches showed PC can reduce oxidative stress, increase epithelial barrier integrity, and decrease intestinal inflammation [13, 14]. PC raises antioxidant SOD and GPX via the increase of the Nrf2/Keap1 ratio, displaying the potential to counterbalance oxidative stress and inflammation in intestinal cells, which improves the insulin sensitivity, and then ameliorates lipid and glucose homeostasis [15]. PC can antagonize oxidative damage by activating the Nrf2 signaling pathway and then protect rat myoblast H9C2 cells against hypoxia/reoxygenation-induced oxidative stress [16]. PC activated Nrf2 translocation and prevented oxidative stress-related intestinal injury and gut pathologies to protect the human colonic cells. Our previous studies also showed that PC-activated Nrf2 then protects epithelial cells from zearalenone-induced oxidative stress and apoptosis [17, 18]. However, the mechanism of how PC activates the Nrf2/ARE signaling pathway is still unknown. This study is aimed at investigating how PC activates the Nrf2 signaling pathway to reduce oxidative stress injury in MODE-K cells.

## 2. Materials and Methods

### 2.1. Chemicals and Drugs

PC, DMSO (Beijing, Solarbio), RPMI 1640, FBS (Israel BI), CCK-8 kit (Beijing, TransGen Biotech), cycloheximide (CHX) (American Sigma), MG132 (Selleck), and MODE-K cells were purchased from Shanghai Guandao Biotechnology Company. Primary and secondary antibodies used in experiments, specifically anti-Nrf2, anti-Ub, anti-*β*-actin, anti-HO-1, anti-Keap1, anti-BACH1, anti-NQO1 (US CST), anti-GAPDH (Suzhou Ruiying), goat anti-rabbit, and goat anti-mouse secondary antibodies, were all from Abcam, USA.

### 2.2. Cell Culture

MODE-K cells were cultured using RPMI1640+10% FBS, take logarithmic growth phase cells and the cell, and the suspension was evenly mixed and then uniformly seeded into a 96-well plate at a cell concentration of 1 × 10^4^ cells/mL and cultured at 37°C, 5% CO_2_. Cell proliferation assays were performed with CCK-8 according to the manufacturer's instructions. The concentration of PC was 0, 10, 20, 30, 40, 60, 80, and 100 *μ*g/mL, and each group had four replicates. After the addition of PC, 10 *μ*L CCK-8 solution was added to each well at 6, 12, 24, and 48 h. The cells were incubated for 1-4 h, and the OD value was measured at 450 nm with a microplate reader. By calculating the relative cell viability of MODE-K cells, the safe concentration of PC for MODE-K cells was determined.

### 2.3. Western Blotting

Cells were first treated with 0, 1, 2.5, and 5 *μ*g/mL PC for 24 h. The effects of different PC concentrations on the expression of HO-1, NQO1, Nrf2, Keap1, and Bach1 proteins were examined. And then, we used 5 *μ*g/mL PC treat cells for 0, 3, 6, 12, and 24 h, respectively, to detect the effects of different times on HO-1, NQO1, Nrf2, Keap1, and Bach1 protein expressions. Three replicates in each group were used.

According to the experimental grouping (experimental group: after 24 hours of intervention with 5 *μ*g/mL PC, 100 *μ*g/mL CHX was applied for 1 h; control group: after 24 hours of culture in serum-free medium, 100 *μ*g/mL CHX was used for 1 h), the intervention was used for the indicated amount of time, and total cell proteins were extracted from the cells at 0, 15, 30, 45, and 60 minutes (according to the literature review, CHX concentration is 100 *μ*g/mL) [2, 19]. After modeling was complete, the total cell protein was extracted. The CHX master solvent is DMSO, and the working solution solvent is RPMI1640.

Cell proteins were separated by sodium dodecyl sulfate-polyacrylamide gel electrophoresis (SDS-PAGE) (Nrf2, Keap1, Bach1 (7.5%), and HO-1, NQO1 (12.5%)) and transferred to PVDF membranes, closed with 5% skim milk. The antibody was diluted with 5% BSA. The PVDF membranes were incubated with anti-Nrf2 (1 : 1000), anti-*β*-actin (1 : 1000), anti-HO-1 (1 : 1000), anti-Keap1 (1 : 1000), and anti-Bach1 antibodies (1 : 1000), overnight incubation of primary antibody. Then, HRP-conjugated secondary antibodies against rabbit IgG (Nrf2, HO-1, Keap1, *β*-actin), and mouse IgG (Bach1, NQO1) were used for detection. Grayscale values of protein bands were analyzed using ImageJ software, protein expression = (target protein gray value − target background gray value)/(internal reference protein gray value − internal reference background gray value).

### 2.4. Immunoprecipitation

The control group, 5 *μ*g/mL PC group, 10 *μ*M MG132 group, and 5 *μ*g/mL PC+10 *μ*M MG132 group were applied for 4 hours, and each group had three repetitions [20–25]. Cell lysates were immunoprecipitated with an anti-Nrf2 antibody, followed by immunoblotting the precipitated proteins with an anti-Ub antibody.

### 2.5. Immunofluorescence

Time gradient and concentration gradient models of PC were established, with three replicates in each group. Then, 4% paraformaldehyde fixative solution was added, and cells were fixed at 25°C for 30 minutes, and then, the fixative was removed. The cell membranes were permeabilized with cold formaldehyde at 25°C for 10 min and then blocked with 5% BSA at 25°C for 2 h at room temperature. After the blocking solution was removed, the Nrf2 antibody (1 : 100) was added to the wells and incubated overnight at 4°C. Then, a secondary antibody, which was fluorescently labeled with FITC (1 : 100) was added and incubated for 60 min in the dark; after the secondary antibody was removed, cells were washed 3 times with PBS, 800 *μ*L of DAPI was added to each well for 5 min, and photos were captured by a fluorescence microscope.

### 2.6. Dual-Luciferase Detection of ARE Gene Activity

Gene synthesis and vector construction were as follows: the NQO1 promoter (1113 bp) was identified, synthesized, and inserted into the pGL3-basic vector. Cell transfection was performed according for GenXPIII Gene Transfection Reagent (ProbeGene CB043). The activity of firefly and Renilla luciferase was detected according to the operation steps of the Dual-Luciferase® Reporter Assay System.

### 2.7. Analysis

SPSS 21.0 (IBM, Almon, NY, USA) software was used for data analysis, and the LSD method was used for multiple comparisons. The test results are expressed as the mean ± standard deviation of three independent tests. *P* < 0.05 and *P* < 0.01 indicate the differences between the test groups.

## 3. Results

### 3.1. Effect of PC on the Relative Survival Rate of MODE-K Cells

**Figure 1:**
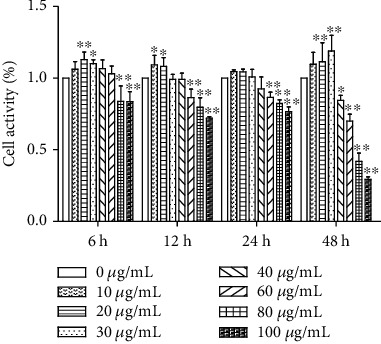
Effects of PC on the viability of MODE-K cells. All tests were repeated at least three times. “∗” represents *P* < 0.05 compared with the control group, and “∗∗” represents *P* < 0.01 compared with the control group.

After PC treatment, the relative survival rate of MODE-K cells gradually increased as the concentration of PC increased to 20 *μ*g/mL; when PC concentration was greater than 20 *μ*g/mL, cell growth was inhibited. Those results indicate that PC is protective against cells within a certain concentration range. When the PC concentration exceeded this range, it had a specific toxic effect on cells and inhibited cell growth. The half-inhibitory concentration of PC on MODE-K cells was IC_50_ = 80 *μ*g/mL, and the safe concentration for MODE-K cells was less than 20 *μ*g/mL (Figure 1). The concentrations of PC we choose for our research were 1 *μ*g/mL, 2.5 *μ*g/mL, and 5 *μ*g/mL, and treat time was 24 h.

### 3.2. PC Affects the Localization of Nrf2 and ARE Gene Activity in MODE-K Cells

**Figure 2:**
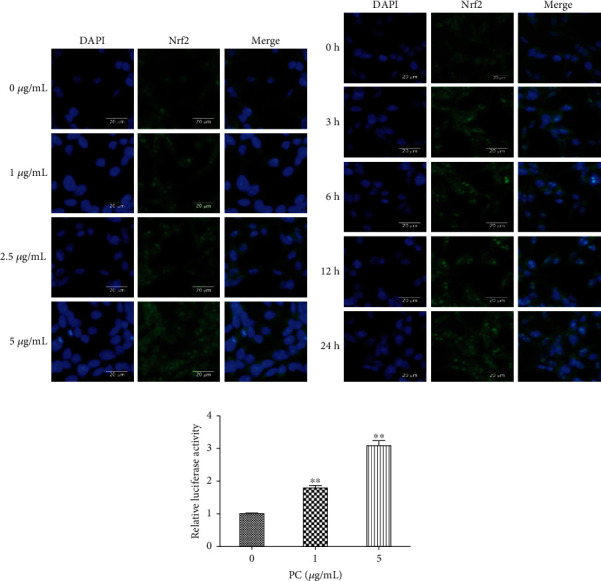
Localization of the Nrf2 protein and ARE gene activity in MODE-K cells with PC-induced concentrations and time gradients. (a) Cells were treated with 0, 1, 2.5, and 5 *μ*g/mL PC for 24 h (40x). (b) Cells were treated with 5 *μ*g/mL PC for 0, 3, 6, 12, and 24 h (40x). (c) Effects of PC on ARE gene activity in MODE-K cells. All tests were repeated at least three times. “∗” represents *P* < 0.05 compared with the control group, and “∗∗” represents *P* < 0.01 compared with the control group.

Results indicated that the increased fluorescence intensity of the Nrf2 protein was observed with increasing PC concentration, and the Nrf2 protein was mainly present in the intranuclear region of the cells. A time gradient could be observed with a significant increase in fluorescence intensity compared to the control group, and the Nrf2 protein was mainly present in the nucleus. PC-induced expression of the Nrf2 protein in MODE-K cells could be observed with green fluorescence throughout the cells but with highlighted sites in the nucleus (Figures 2(a) and 2(b)).

As shown in Figure 2(c), compared to the control group, 1 *μ*g/mL PC significantly increased the fluorescence intensity of the ARE element of MODE-K cells (*P* < 0.01); compared with 1 *μ*g/mL PC, 5 *μ*g/mL PC significantly increased the fluorescence intensity of ARE elements in MODE-K cells (*P* < 0.01). PC significantly increased the activity of ARE gene in MODE-K cells.

### 3.3. Effect of PC on Nrf2 and Bach1 Protein Expression in MODE-K Cells

**Figure 3:**
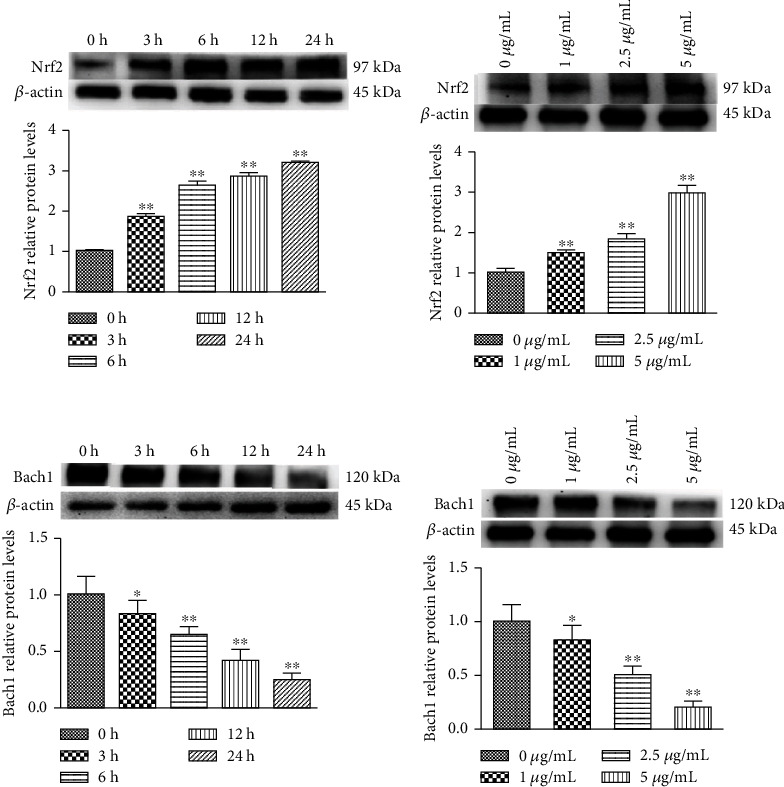
Concentration gradient PC-induced relative protein levels of Nrf2 and Bach1 in MODE-K cells. (a and c) Cells were treated with 5 *μ*g/mL PC for 0, 3, 6, 12, and 24 h. (b and d) Cells were treated with 0, 1, 2.5, and 5 *μ*g/mL PC for 24 h. All tests were repeated at least three times. “∗” represents *P* < 0.05 compared with the control group, and “∗∗” represents *P* < 0.01 compared with the control group.

When treated the cells with 5 *μ*g/mL PC, the protein expression of Nrf2 increased with the time increased. Compared with the 0 h group, the Nrf2 protein expression level of PC at 3, 6, 12, and 24 h increased significantly (*P* < 0.01) (Figure 3(a)). As shown in Figure 3(b), compared with the 0 *μ*g/mL PC group, the expression of the Nrf2 protein in MODE-K cells increased significantly in 1, 2.5, and 5 *μ*g/mL PC groups (*P* < 0.01). As shown in Figure 3(c), compared with the 0 h group, 5 *μ*g/mL PC significantly reduced the protein expression of Bach1 with the time increased (*P* < 0.05), and the reduction in Bach1 protein expression was time-dependent. As shown in Figure 3(d), in the case of 1, 2.5, and 5 *μ*g/mL PC induction, the expression of Bach1 protein in MODE-K cells decreased with increasing concentration in a concentration-dependent manner.

### 3.4. Effect of PC on NQO1 and HO-1 Protein Expression in MODE-K Cells

**Figure 4:**
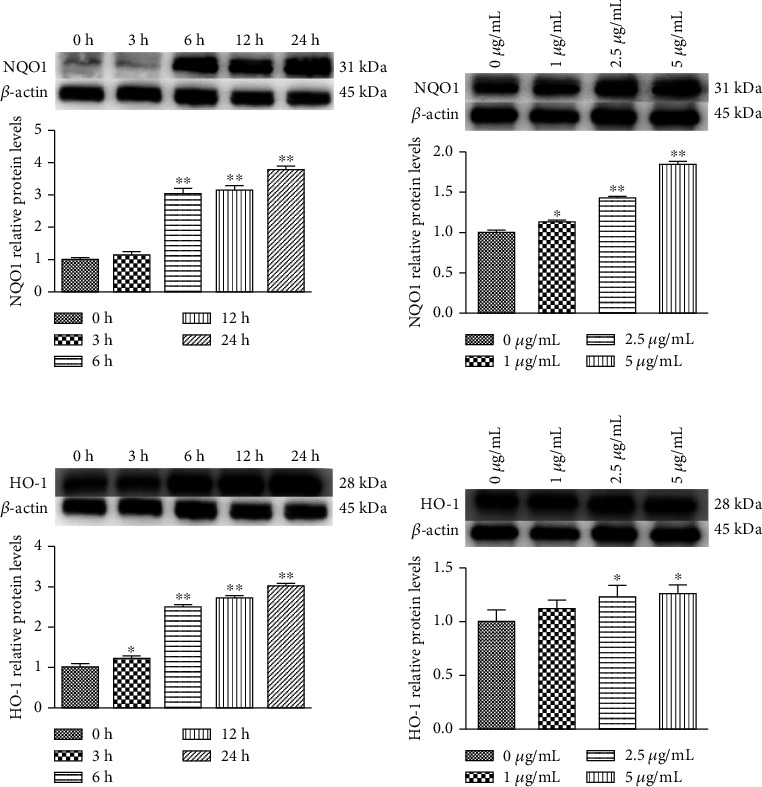
PC-induced relative protein levels of NQO1 and HO-1 in MODE-K cells. (a and c) Cells were treated with 5 *μ*g/mL PC for 0, 3, 6, 12, and 24 h. (b and d) Cells were treated with 0, 1, 2.5, and 5 *μ*g/mL PC for 24 h. All tests were repeated at least three times. “∗” represents *P* < 0.05 compared with control group, and “∗∗” represents *P* < 0.01 compared with control group.

As shown in Figures 4(a) and 4(c), NQO1 and HO-1 protein expressions increased with time when treated the cells with 5 *μ*g/mL PC. Both HO-1 and NQO1 protein expression trends were consistent and time-dependent. As shown in Figures 4(b) and 4(d), NQO1 and HO-1 protein expressions in MODE-K cells were increased with increasing PC concentration. Both HO-1 and NQO1 protein expression trends were consistent, and both were concentration-dependent.

### 3.5. Effect of PC on Keap1 Protein Expression in MODE-K Cells

**Figure 5:**
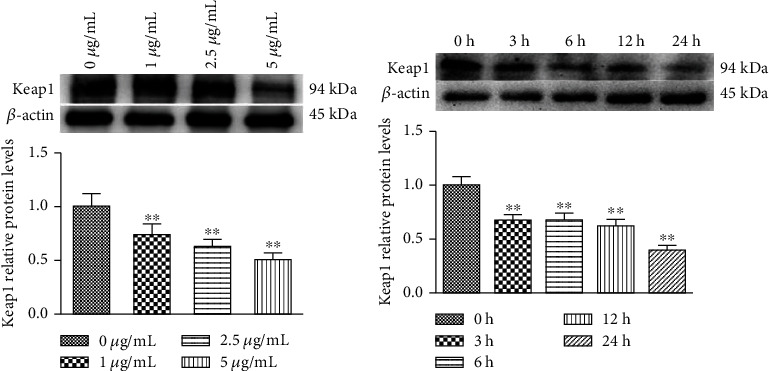
PC-induced relative protein levels of Keap1 in MODE-K cells. (a) Cells were treated with 0, 1, 2.5, and 5 *μ*g/mL PC for 24 h. All tests were repeated at least three times. (b) Cells were treated with 5 *μ*g/mL PC for 0, 3, 6, 12, and 24 h. All tests were repeated at least three times. “∗” represents *P* < 0.05 compared with the control group, and “∗∗” represents *P* < 0.01 compared with the control group.

The results are shown in Figure 5(a); with the concentration of PC increased, Keap1 protein expression decreased significantly compared with the 0 *μ*g/mL PC group. As shown in Figure 5(b), 5 *μ*g/mL PC significantly decreased Keap1 protein expression in MODE-K cells after being treated 3 h to 24 h, compared to the control group.

### 3.6. Stability and Ubiquitination of the Nrf2 Protein in MODE-K Cells

**Figure 6:**
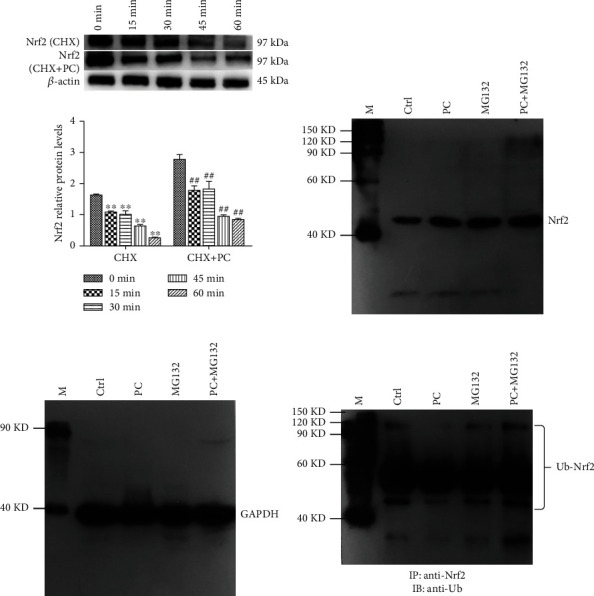
PC-induced Nrf2 protein stability and ubiquitination in MOKE-K cells. (a) Intervention with 5 *μ*g/mL PC for 0, 15, 30, 45, and 60 min and the control group for 24 h (100 *μ*g/mL CHX for 1 h). All tests were repeated at least three times. (b–d) PC-induced Nrf2 relative protein levels in MODE-K cells. “M” is Easysee protein marker, “Ctrl” is the control group, and GAPDH is the internal control. “∗” represents *P* < 0.05 compared with the CHX control group, “∗∗” represents *P* < 0.01 compared with the CHX+PC control group. “#” represents *P* < 0.05 compared with the CHX control group, and “##” represents *P* < 0.01 compared with the CHX+PC control group.

As shown in Figure 6(a), the protein synthesis inhibitor (CHX) blocked Nrf2 protein expression, and the total amount of protein decreased with treated time too. Compared with the CHX group alone, the Nrf2 protein expression in the CHX+PC group was higher and degraded at a lower rate. These results show that PC can increase the stability of the Nrf2 protein, slow the degradation of Nrf2 protein, and extend the half-life of Nrf2 protein. As shown in Figures 6(b) and 6(c), both 5 *μ*g/mL PC and 10 *μ*M MG132 (proteasome inhibitor) can increase Nrf2 protein expression compared with the control group, and there was no significant difference between PC+MG-132 group and MG132 group. As shown in Figure 6(d), cells treated with PC decreased the ubiquitination level, and treatment with MG132 also decreased the ubiquitination level of the Nrf2 protein compared with the control group. The experimental results suggest that PC increases Nrf2 protein levels and activates the Nrf2/ARE signaling pathway by inhibiting Nrf2 protein ubiquitination and degradation.

## 4. Discussion

Many reports suggest that the activation of Nrf2/ARE signaling pathway is mainly due to the increased stability of the Nrf2 protein, leading to a large number of Nrf2 entering into nucleus and binding to ARE elements, which in turn activates a series of downstream antioxidants and phase detoxification enzymes that exert antioxidant and cell-protective effects [26–29]. Since natural antioxidants are widely found in food, most toxic substances in the environment damage the body through the digestive system, and the first site which absorbs the toxic substances is the duodenum. The MODE-K cell line is reliable in vitro model similar to the human/mouse duodenum that can well mimic the cytoprotective defense against Nrf2-mediated oxidative stress, and MODE-K cells were chosen for study [30, 31].

First, we tested the distribution of the Nrf2 protein in MODE-K cells in the presence of PC. Our results show that Nrf2 protein in the nucleus increased with increasing PC concentration. Under normal physiological conditions, the Bach1 protein forms dimers with small Maf proteins and binds to ARE elements, thereby inhibiting the activation of the Nrf2/ARE signaling pathway and the expression of downstream genes [32]. We used PC-induced activation of the Nrf2/ARE signaling pathway after Bach1 and Nrf2 protein expression to verify the relationship between Bach1 protein and Nrf2 protein competitive inhibition. The results showed that the expression of the Nrf2 protein increased with increasing PC concentration, while the expression of Bach1 protein showed the opposite trend which decreased with increasing PC concentration. These results suggest that Nrf2 and Bach1 are competitively inhibited in PC-induced MODE-K cells. PC can increase Nrf2 protein expression and decrease Bach1 protein expression. Competitive inhibition of the Bach1 protein and Nrf2 protein is also an interesting area in studying the activation mechanism of the Nrf2 protein signaling pathway [28]. The main reason which increased HO-1 and NQO1 expression might be the activation of the Nrf2/ARE signaling pathway [14, 33]. To determine whether PC can activate the Nrf2/ARE signaling pathway in MODE-K cells, we examined the expression of HO-1 and NQO1 proteins. The Bach1 protein expression was opposite to the expression trend of HO-1 and NQO1 proteins. As an inhibitor of the Nrf2/ARE signaling pathway, the expression of Bach1 protein decreased leading to the expression of HO-1 and NQO1 protein increased. Activation of the Nrf2/ARE signaling pathway could regulate the expression of phase II detoxification enzymes, and antioxidant enzymes, such as HO-1 and NQO1, are key indicators involved in antioxidant stress [34]. Related studies have shown that grape seed PC can increase HO-1 expression in human embryonic kidney cells through the Nrf2 signaling pathway [8]. PC can increase the expression of NQO1 protein in A549 cells by activating the Nrf2 signaling pathway [14]. The ARE element is located in the promoter region of genes downstream of the Nrf2 signaling pathway, and both Bach1 and Nrf2 can bind the ARE element to regulate the expression of HO-1 and other antioxidant proteins [32]. We tested the ARE gene activity in PC-induced MODE-K cells. The results showed that PC increased the gene activity of ARE elements in a concentration-dependent manner; the result is consistent with our previous results on HO-1 and NQO1 protein expression and confirms that PC can activate the Nrf2/ARE signaling pathway in MODE-K cells, thereby allowing the Nrf2 protein entered the nucleus and bind to the ARE element to activate the Nrf2 signaling pathway and regulate the downstream expression of the HO-1 and NQO1 proteins. It has been shown that the Nrf2 protein entering the nucleus can form a dimer with a small Maf and bind to the ARE to activate downstream gene expression [15]. Bach1 forms a Bach1-Maf heterodimer with Maf in the nucleus and binding to ARE. Bach1 can competitively inhibits with Nrf2 which bind to ARE elements, so the cell is unable to initiate the Nrf2 signaling pathway and may inhibit the transcription of many oxidative stress response genes, such as HO-1 and NQO1 [33]. A large number of Nrf2 enter into the nucleus and bind to the antioxidant element ARE and then activate subsequent antioxidant enzymes and phase II detoxification enzymes [35]. On the other hand, Bach1 acts as an inhibitor of the Nrf2/ARE pathway, and Bach1 can negatively regulate antioxidant genes such as HO-1 and NQO1, which is consistent with our experimental results. Our results also confirm that PC is an activator of the Nrf2/ARE signaling pathway.

Keap1 mediates Keap1-dependent Nrf2 protein ubiquitination degradation and negatively regulates Nrf2 activity [36]. Under normal physiological conditions, Nrf2 protein is located in the cytoplasm and binds to Keap1, and the Nrf2 protein can be degraded via the ubiquitin-proteasome pathway and decreased Nrf2 protein levels. When cells are stimulated by free radical, Nrf2 is released from Keap1 and entered the nucleus, which bind to ARE and then activates the Nrf2/ARE signaling pathway [37]. To verify the role of PC on Keap1 and Nrf2 protein expression, our results showed that Keap1 protein expression decreased with increasing concentration and time. Keap1 has been shown to regulate the stability of the Nrf2 protein [29], and we have demonstrated that PC induction increases Nrf2 protein expression. To further determine the reason for the increased Nrf2 protein expression, we determined the Nrf2 protein half-life using cycloheximide (CHX). In the CHX group, the degradation rate of the Nrf2 protein decreased to approximately 50% in 30 min, and it degraded approximately 50% in 45 min in the PC+CHX group. It was demonstrated that PC could prolong the half-life of the Nrf2 protein and slow its degradation, and PC increased the stability of the Nrf2 protein in MODE-K cells. The Nrf2 protein can be degraded through the ubiquitin-proteasome pathway [24]. To further verify the possible effect of PC on the ubiquitin-proteasome pathway of the Nrf2 protein and determine whether PC inhibits the ubiquitination of the Nrf2 protein, the interaction between the Nrf2 protein and ubiquitin protein was investigated. Both PC and MG132 alone increased the expression of the Nrf2 protein. There was no significant difference between PC+MG132 and MG132 group on Nrf2 protein expression. The test results showed that PC could inhibit the ubiquitin-proteasome degradation of the Nrf2 protein, thus reducing the degradation rate of the Nrf2 protein and improving the stability of the Nrf2 protein. The binding of the Nrf2 protein to ubiquitin was reduced by PC and increased by MG132. The inhibitory effect of MG132 on the ubiquitin-proteasome mainly blocked the proteasomal pathway, which had no effect on the binding of the Nrf2 protein to ubiquitin molecules. And the mechanism of PC was different from that of MG132, in which PC mainly inhibited the binding of Nrf2 and ubiquitin molecules and then reduced the ubiquitin-proteasome degradation of the Nrf2 protein. PC increases the accumulation of the Nrf2 protein by decreasing the ubiquitination and degradation of the Nrf2 protein, which further activates the Nrf2/ARE signaling pathway. It was shown that some antioxidants, such as PC, might activate the Nrf2/ARE signaling pathway mainly due to the increased stability of the Nrf2 protein, which allows the Nrf2 protein to accumulate in cells [28]. Our results also showed the effect of PC on the Nrf2 protein is the same as that of Nrf2 inducers such as tBHQ and oridonin, indicating that PC is a potent Nrf2 activator [21].

The mechanism of activation of the Nrf2/ARE signaling pathway is mainly an increase in the total amount of the Nrf2 protein [21, 38, 39]. PC reduces the total amount of the Keap1 protein, decreases Nrf2 ubiquitination, and inhibits degradation of the ubiquitin-proteasome pathway of the Nrf2 protein, thus increasing the accumulation of the Nrf2 protein in the cell and activating the Nrf2/ARE signaling pathway. Keap1 protein can negatively regulate Nrf2 protein activity, and Bach1 protein competitively inhibits Nrf2 protein binding to ARE. PC decreased Keap1 protein expression and Nrf2 ubiquitination levels, increased Nrf2 protein stability, and increased downstream HO-1 and NQO1 protein expressions in MODE-K cells, thus indicating that inhibition of Keap1-dependent Nrf2 ubiquitination levels can increase the expression of antioxidant genes whose stability is dependent on Nrf2 activation, in line with previous findings [21]. We demonstrated that ubiquitination of the Nrf2 protein in MODE-K cells was reduced due to PC-mediated inhibition of the Keap1 protein-dependent degradation of the ubiquitin-proteasome pathway of the Nrf2 protein, inhibition of the ubiquitin-proteasome pathway of the Nrf2 protein degradation, and increased half-life of the Nrf2 protein. Then, it leads to the accumulation of Nrf2 in cells and induces the activation of the Nrf2/ARE signaling pathway. However, our study did not demonstrate non-Keap1-dependent Nrf2 ubiquitination degradation without PC inhibition, and whether PC can activate the Nrf2 signaling pathway by inhibiting Keap1-independent Nrf2 ubiquitination degradation remains to be further investigated.

We investigated the mechanism of PC-induced activation of the Nrf2 signaling pathway in MODE-K cells. The PC-mediated activation of the Nrf2 pathway, which induces the expression of its downstream genes and thus activates cytoprotective effects, likely explains the potent antioxidant, anti-inflammatory, and anticancer properties of these food-derived antioxidants [40, 41]. The use of plant-derived natural substances to trigger the expression of downstream protective genes and other intracellular defense systems through Nrf2 activation provides greater advantages for the treatment and prevention of proposed diseases [42, 43].

## 5. Conclusion

**Figure 7:**
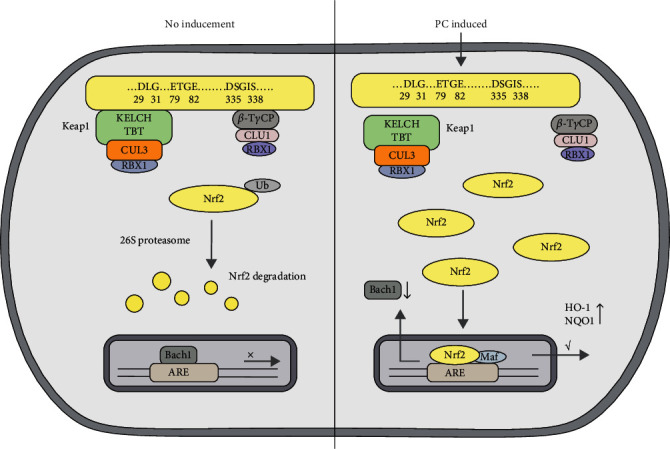
PC activates the Nrf2/ARE signaling pathway in MODE-K cells.

We demonstrated that PC could activate the Nrf2/ARE signaling pathway in intestinal epithelial cells by inhibiting Keap1-dependent ubiquitination and Nrf2 protein degradation. PC decreases the expression of the Keap1 protein, decreases the ubiquitination of the Nrf2 protein, inhibits the ubiquitination degradation of the Nrf2, and increases the stability of the Nrf2 protein, increasing Nrf2 entering into the nucleus, competes with Bach1 to bind with ARE, which in turn initiates the Nrf2/ARE signaling pathway, regulates downstream antioxidant enzymes and phase II detoxification enzymes and other related genes, and initiates cytoprotection (Figure 7).

## Data Availability

All the data used to support the findings of this study are included within the article.
